# In Vivo Passive Sampling Implantation in Fish for Monitoring of PAHs: Calibration and Kinetics

**DOI:** 10.3390/jox16010032

**Published:** 2026-02-10

**Authors:** Jhon Fredy Narváez Valderrama, Juan José García Londoño, Daniel Gil Ramírez, Clara S. Arias-Monsalve, Jorge L. Gallego

**Affiliations:** 1 Grupo de Investigación Ingeniar, Facultad de Ingenierías, Corporación Universitaria Remington, Calle 51 No. 51-27, Medellín 050012, Colombia; 2 Grupo de Investigación Materialografía, Transición Energética y Ambiente (MATREA), Facultad de Ingenierías, Corporación Universitaria Remington, Calle 51 No. 51-27, Medellín 050012, Colombia; juan.garcia.3750@miremington.edu.co; 3 Facultad de Ingeniería, Universidad de la Sabana, Chía 250017, Colombia; danielh.gil@udea.edu.co; 4 Grupo en Salud Familiar y Comunitaria, Facultad de Ciencias de la Salud, Corporación Universitaria Remington, Calle 51 No. 51-27, Medellín 050012, Colombia; clara.arias@uniremington.edu.co; 5 Biodiversity, Biotechnology and Bioengineering Research Group GRINBIO, Department of Engineering, University of Medellin, Medellín 050026, Colombia; jlgallego@udemedellin.edu.co

**Keywords:** bioaccumulation, dissipation modelling, passive sampling, silicone rubber membranes (SRMs), polycyclic aromatic hydrocarbons (PAHs)

## Abstract

Polycyclic aromatic hydrocarbons (PAHs) can enter water bodies and bioaccumulate in fish, leading to biomagnification; therefore, their monitoring is necessary. Passive sampling is easy to handle and shows potential for this purpose. However, studies in vivo are scarce, and kinetic parameters governing analyte partitioning between tissue and samplers remain poorly characterized. In this study, the silicone rubber membranes (SRMs) were exposed to fish fillet from common carp (*Cyprinus carpio*) to determine bioaccumulation parameters based on dissipation modelling using performance reference compounds (PRCs). The SRM was implanted in vivo in fish, and the dissipated PRCs were measured and applied to a mono-compartmental model. The results in fish fillet showed a pseudo-first kinetic order, and the plateau was attained at a time > 30 h. However, the equilibrium may not be ensured because of the low lipid fraction (*fl*) in fish (4.5%), which could lead to a local saturation of the tissue in contact with the SRM. The ratio between elimination and uptake constants (*K_e_*/*K_u_*) showed faster PAHs–SRM sorption than PAHs-fish tissue sorption (200 times); thus, fish with low *fl* will lead to faster SRM sorption. By contrast, in fish with higher *fl*, the long-term exposures will be necessary. The percentage of released deuterated PAHs from SRM during in vivo fish exposure was 1.6 times higher than that observed in the fish fillet, indicating an active clearance process. Therefore, during implantation, the rate of clearance and the *fl* should be considered to ensure detectable levels for applying the integrative equation based on dissipation modelling.

## 1. Introduction

Aquatic organisms are exposed to low levels of hydrophobic organic compounds (HOCs), which are continuously discharged from different anthropogenic sources [1]. Those substances are biomagnified in the trophic chain and can reach the human body as a final receptor through dietary exposure [2]. For instance, some HOCs such as DDT, endosulfan, polychlorinated biphenyls (PCBs), and especially polycyclic aromatic hydrocarbons (PAHs) have been detected in fish tissue at levels ranging from 0.05 to 72.53 ng g^−1^ wet weight [3]. However, the concentration of HOCs in fish tissue is highly variable and strongly influenced by the lipid fraction (*fl*), as it enhances the contaminant sorption process through lipophilic interactions [4]. Thereby, the bioaccumulation factors (BAFs) are positively correlated with fish *fl*, which must be considered when interpreting tissue burden data.

PAHs are of ecotoxicological relevance because they are ubiquitous contaminants distributed across multiple environmental compartments, including air, water, soil, and biota [5,6]. They can reach waterbodies by runoff and atmospheric deposition [7], leading to their bioaccumulation in aquatic organisms and humans. We have previously found that PAH congeners, such as anthracene (ANT), fluoranthene (FLU), pyrene (PYR), and benzo[a]pyrene (BaP), induce changes in reproductive hormone levels during in vitro assessment [8], suggesting potential endocrine-related effects in aquatic species and humans [9,10].

Occurrence of PAHs in water and organisms is often reported at trace levels; however, their metabolic fate, chronic effects, and long-term internal exposure dynamics remain insufficiently characterized in different species [11]. Although PAH bioaccumulation in aquatic organisms has been widely investigated, the interpretation of tissue concentrations is constrained by several biological and analytical limitations. In particular, rapid metabolic transformation, elimination through multiple physiological pathways, and strong partitioning into lipid-rich compartments can substantially reduce the fraction of parent PAHs available for extraction and quantification, especially the remaining fractions which are strongly bonded to *fl* in fish [12]. As a result, measured concentrations in biological tissues may not fully represent total internal PAH burdens or long-term exposure, restricting the assessment and comparison of bioaccumulation across studies.

Some emerging alternatives have been applied for biomonitoring, such as polymer membrane implantation as a passive sampling methodology in biota. For instance, O’Connell implanted some silicone rubber membranes (SRMs) in dorsal and ventral locations of female ICR mice to measure chemical bioaccumulation, such as p,p′-DDE, and PCB118 ratios, in adipose tissue by intraperitoneal injection [13]. The SRM may play an important role as a sink for HOCs, and then, after their removal from implanted species, many uptake substances are analyzed [13]. Therefore, O’Connell and colleagues predicted the adipose concentrations for a wide range of pollutants in human tissue [13]. Additionally, Rusina and colleagues have applied equilibrium passive sampling for a fish tissue burden study using performance reference compounds (PRCs) [14]. The equilibrium may be reached between lipid-rich tissues and the polymer left in contact with fish at a specific time (*t*) by diffusion mechanisms, according to Fick’s law. However, the estimation of the equilibrium concentrations should consider the percentage of lipids, the type of tissue near the sampler surface, and even the metabolic processes. Although partition coefficients have been estimated between *fl* and passive sampler polymers (PSPs), the metabolic processes and active diffusion in vivo assays have not been performed. Additionally, previous reports indicate that in vivo passive sampling has the potential to be applied to organisms with lower lipid content because fugacity may be an important issue for biomagnification processes in the trophic chain [15].

In this research, we carried out an in vivo passive sampling implantation of SRM in common carp (*Cyprinus carpio*) for PAH bioaccumulation assessment. Initially, the method was tested in fish fillets for estimating kinetic parameters. Thereby, those fillets were led in contact with SRM previously spiked with deuterated PAHs used as PRCs to analyze kinetic interchange processes. Lately, the SRM implantation was scaled up in vivo to establish the bioaccumulation of PAHs in carp, applying the dissipation modelling of PRC. The dissipated fraction of PRC was applied in a single-compartment bioaccumulation model for *Cyprinus carpio*.

## 2. Materials and Methods

### 2.1. Materials

The silicone rubber membranes (SRMs) were supplied by Altec Laboratory (Victoria St Austell, UK), with a thickness of 0.5 mm. The deuterated PAHs standards, fluoranthene (FLU) and pyrene (PYR), were purchased from Sigma-Aldrich, Inc, St. Louis, MO, USA (Purity > 99.5%). Those deuterated substances avoid the cross-contamination, which may reduce the signal area during chromatography analysis and thus the levels reported during experiments. The solvents, including hexane, methanol, and acetonitrile, were supplied by PanReac AppliChem, Darmstadt, Germany; while the Milli-Q water was obtained through Thermo Scientific^®^ Barnstead (Thermo Fisher Scientific Inc., Waltham, MA, USA; Purity > 99.5% for pesticide analysis). Finally, the 2-Phenoxyethanol (CAS # 122-99-6) was supplied by Sigma Aldrich.

### 2.2. SRM Preparation and Clean-Up

The SRM sheets were cut into 1.5 × 5 cm strips with a total surface area of 15 cm^2^ (568–641 mg)–(0.375 mL). For clean-up, SRM sheets were pre-extracted by dialysis with 100 mL of methanol to remove non-polymerized monomers and impurities, respectively, for 24 h and subsequently with 100 mL of hexane to remove lipophilic pollutants [14]. Finally, the SRM sheets were sterilized in an autoclave.

### 2.3. Spiking SRM with Deuterated PAHs Used as PRCs

A total of 20 SRM sheets were spiked in a batch experiment with low levels of deuterated PAH congeners to avoid mortality in exposed fish, specifically FLU and PYR. Those substances show lower fugacity and higher fish biotransformation, which may reduce the transference to SRM due to previous fish exposure [15]. The methodology applied was followed according to previous reports by Foppe Smedes and coworkers and modified [16]. Details are provided in Supplementary Material (See Figure S1a,b). Overall, 20 SRM were introduced inside an amber flask with 15 mL of methanol, and 100 μL stock solution of deuterated PAHs was spiked (11.8 ppm—FLU and 7.3 ppm—PYR). The amber flask was shaken (130 rpm) for 5 days under stepwise addition of water, ending in ratios of 50:50 methanol: water.

### 2.4. SRM Extraction and Analysis by GC/MS

After spiking, the individual SRMs were introduced into amber flasks, and 10 mL of hexane was added. Then, the samples were shaken for 24 h, and the extraction was carried out twice. The final volume of hexane was reduced by a Heidolph Rotary Evaporator Hei-VAP to 1 mL, which was adjusted using nitrogen gas.

The analysis of deuterated FLU and PYR was carried out by Thermo Scientific Ultra TRACE GC-DSQII, electron-impact, and a single quadrupole for mass spectrum confirmation of PAHs. The congeners were separated on a Rtx 5sil-MS (30 m × 0.25 mm I.D., 0.25 m film thickness) capillary column from Restek using helium gas as a carrier at a flow rate of 1 mL min^−1^. At first, the column was kept for 1 min at 50 °C; second, the temperature increased to 320 °C at a rate of 25 °C min^−1^ and held for 1 min. The injector and interface temperature were maintained at 270 °C, and the source temperature at 250 °C. The injection volume was 1 µL. The instrument was operated in Selection Ion Monitoring (SIM) for quantification purposes, while the SCAN mode was operated for qualitative purposes.

### 2.5. The SRM–Fish Fillet Tissue Partition

The fish fillet was cut into portions with an average weight of 54.26 ± 7.39 g. Then, the fillets were butterflied into similar pieces, and the spiked SRM was introduced between the two parts of each small fillet, creating a “sandwich”. Additionally, a fillet was exposed to the SRM blank. The fish samples were taken at 12, 24, 48, 72, 90, and 120 h per duplicate. For each of them, the SRM was removed and extracted as described before. Additionally, 5 g of fish fillet was weighed in a 50 mL polypropylene centrifuge tube for extraction to determine the lipid content according to Peter Tölgyessy. In total, 5 mL of the acetone and ethyl acetate mixture (6:4, *v*/*v*) was added. The mixture was shaken at 1200 rpm for 3 min. Then, approximately 2 g of anhydrous magnesium sulphate (MgSO_4_) and 0.5 g of NaCl were added, and the tube was shaken for 3 min. Thereby, the mixture was centrifuged for 5 min at 5000 rpm. An aliquot of the upper organic phase was transferred into a pre-weighed glass tube. The extracts were then dried under nitrogen until constant weight. Final residues were weighed, and the percent of lipid was estimated [17]. Finally, the results were plotted for kinetic analysis.

### 2.6. Calibration of SRM−Tissue Partition by Dissipation Modeling

The pollutants bioaccumulated in fish may diffuse in tissue according to Fick’s first law, and exchange processes between phases follow a first-order kinetics according to Equation (1) [18]. This kinetic order has been previously applied for in vivo passive sampling to measure elimination kinetics in fish:

(1) $$C_{fish}=C_{sm}*e^{k_{us}*t}$$

where *C_fish_* and *C_sm_* are the pollutant concentrations in the fish model and SRM, respectively (ng·mg^−1^). *k_u_* is the uptake constant in time (*t*) in SRM (hours). The interchange and elimination process completes the accumulation factor (AF) in SRM to reach the concentration approach at the steady state, which is the ratio between *C_sm_*/*C_fish_* (also the partition coefficient). Therefore, Equation (2) represents the AF, which is a similar value to the partition coefficient, which will be presented forward.

(2) $$AF=\frac{C_{sm}}{C_{fish}}=\frac{K_{e}}{K_{u}}$$

where *K_e_* is the elimination rate constant, which may be found by the PRC dissipation modelling, applying Equation (3).

(3) $$K_{e}=\;\;\frac{\ln(\frac{No}{Ni})}{t}\;\;\;$$

where *N_o_* and *N_i_* are the initial and final amounts of deuterated PAHs spiked into SRMs; therefore, the *k_u_* may be calculated according to ratios between both kinetic constants in the bioaccumulation study.

Additionally, the concentration of pollutants at steady state in the SRM ($C_{sm\;}^{\infty }$) may be calculated by compounds dissipated in time *t* according to Equation (4) [15].

(4) $$C_{sm}^{\infty }=C_{sm}\frac{No}{No-N_{i}}$$

**Figure 1:**
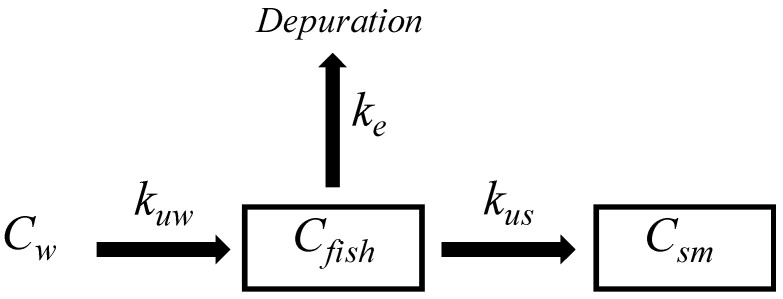
Single-compartment fish model for bioaccumulation and elimination of PAHs.

The bioaccumulation study includes the exchange of pollutants from water to fish according to the uptake kinetic constant (*K_uw_*). The substances may be distributed in organisms according to the single-compartment model, which includes tissue distribution and depuration, which is done according to the elimination constant (*K_e_*). See Figure 1.

When the spiked SRM is implanted in fish tissue, the PRCs may be dissipated into fish tissue according to *K_e_*, and those fractions may be depurated (See Figure 1). Similarly, substances previously bioconcentrated in fish may reach the SRM according to the uptake kinetic constant (*K_us_*), and substances may thereby reach a steady state in both phases (see Equation (5)).

(5) $${C_{sm}=C}_{fish}k_{sf}$$

where the *K_sf_* is the partition constant between fish fillet and SRM. To find the levels of pollutants in fish tissue, the complete interchange–elimination processes could be integrated into Equation (6).

(6) $$C_{fish}=\frac{C_{sm}}{k_{sf}}\;\left(1-e^{-k_{e}.t}\right)$$

### 2.7. Fish In Vivo Bio-Model

To go one step further, in vivo experiments were carried out. The experimental protocol was reviewed and approved by the Institutional Veterinary Ethics Committee of the Faculty of Veterinary Medicine (Acta No. 004-2017, approved in 2017). All procedures complied with the principles of animal welfare, including minimization of pain, stress, and discomfort throughout handling, anesthesia, surgery, and recovery. The surgery was initially performed on dead fish to find the best procedure for surgical incisions and sutures. The vertical cut was done at 2.0 cm, with a depth of around 6 mm, and the internal longitudinal cut was 6 cm. Therefore, the spiked SRM was introduced thoroughly, and the cut was sutured using a suture thread 0.65 mm in diameter. Finally, a bone scan was performed on the fish to see the implantation.

Initially, anesthesia preliminary testing was carried out in five individual fish (*Cyprinus carpio*). During the experiment, the time for induction of anesthesia (min) and the time for recovery (min) were assessed. The anesthesia stage was assessed by the analysis of loss of equilibrium, slow and regular opercular movements, and a loss of spinal reflexes [19,20]. The oxygen was supplied by pumping water through the operculum. More details are presented in the Supplementary Materials (see Figure S7a).

In vivo experiments were carried out in four individual fish (*Cyprinus carpio*) with an approximate weight of 950 to 1050 g (26 to 28 months old). The culture conditions, such as dissolved oxygen, turbidity, temperature, and pH, were under control. More details are presented in the Supplementary Materials (See Figure S6d). They were anesthetized using 2-Phenoxyethanol. For that, individuals were introduced in 16 L of water at concentrations of 0.7 mL L^−1^ (2-Phenoxyethanol), and the time to reach stage 4 (slow opercular rhythm and total loss of balance) was 2.74 s. The stage of anesthesia was assessed using three individual carp.

The fish scales were removed, and a vertical incision was carried out at 2.0 cm near the dorsal fin to introduce the SRM—more details can be found in Supplementary Materials (see Figure S7c). After anesthesia recovery, each fish was introduced into a plastic fishpond, and individual SRMs were extracted and collected at 48 h to remove membranes under ethical protocols. The SRMs were stored in aluminum foil until their analysis by GC/MS.

### 2.8. Equilibrium in SRM and Fish Lipid Fraction (FL) Analysis

The removed SRMs from *Cyprinus carpio* were extracted overnight by dialysis using hexane (See SRM extraction and analysis by GC/MS section) [21]. After dialysis, the hexane was concentrated by rotary evaporator, and fractions were reduced to 1 mL using nitrogen gas, which was analyzed by GC/MS. The deuterated PAHs concentration in fish tissue (*C_fit_*) was estimated by the ratio of the differences between the initial concentrations of PAHs in SRM and the final concentrations in SRM after tissue exposure and the mass of tissue. See the following Equation (7):

(7) $$C_{fit}=\;\frac{\left(initial\;concetrations\;of\;PAHs\;in\;SRM\;-\;\;final\;concentrations\;of\;PAHs\;in\;SRM\right)}{mass\;fish\;tissue\;}$$

### 2.9. Data Analysis

The graphs were plotted using GraphPad Prism 7.0. For case and control statistics, a 2-way ANOVA was applied to find significant differences in groups using the same statistical software.

## 3. Results and Discussion

### 3.1. Analysis by GC/MS

The method for PAH congeners FLU and PYR showed a good performance by GC/MS. More details are presented in the Supplementary Material (See Figure S3). The time retention and validation parameters are presented in Table 1. The sensibility and reproducibility were appropriate for our quantification purpose.

Finally, the homoscedasticity and the *r*^2^ showed good linearity. More details are presented in Supplementary Materials (see Figure S4).

The confirmation of PAH congeners by GC/MS showed the presence of FLU and PYR in all samples at 20.7 min and 21.1 min, respectively. Additionally, the mass spectrum for each compound showed a correlation > 99.9% in the NIST/EPA/NIH Mass Spectral Library—more details can be found in the Supplementary Materials (See Figure S3). Also, the sensitivity of this method showed a LOD lower than 9.5 μg·L^−1^, which is enough for the dissipation analysis of PRC at percentages higher than 80%.

### 3.2. Spiking SRM with PRCs

**Figure 2:**
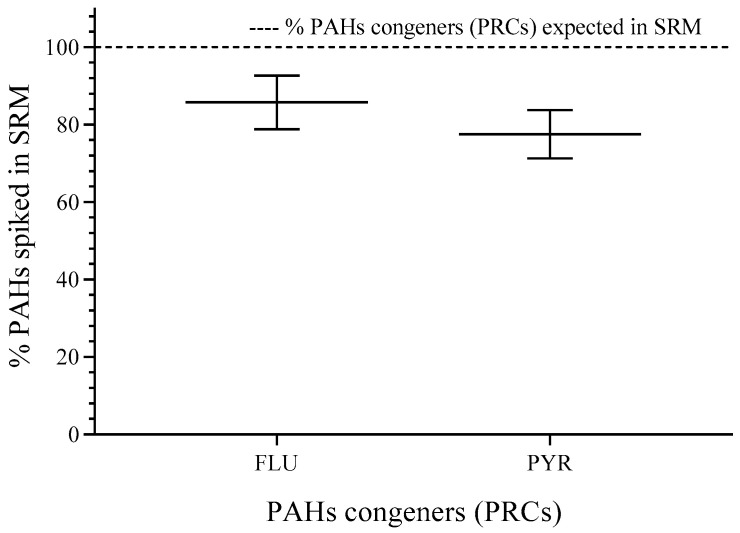
PAH congeners spiked in SRM. RSD for individual PAHs (*n* = 3). FLU (8.1%) and PYR (8.1%). *n* = 3.

The percentage of spiked FLU and PYR showed values higher than 80% (compared to the expected value for FLU—95.3 ng·mg^−1^ and for PYR—58.8 ng·mg^−1^) of the nominal concentration and relative standard deviation (RSD) < 10%. More details can be found in the Supplementary Materials (See Table S1). The weight found for SRM was 620.54 ± 20.44 mg, and the RSD was 3.4%, which was acceptable for experimental error (see Figure 2).

The spiked PAHs (FLU and PYR) show *log K_ow_* higher than 4, which indicates lipophilic properties [22]. Thereby, the interchange from polar spiking solvent to SRM is possible because PAHs show more affinity for non-polar phases [23]. Finally, the concentrations in SRM for each congener applying single calibration curves allowed the mass estimation, which was divided by the average weight for membranes (620.54 ± 20.44 mg), and the levels of deuterated PAHs are reported in Table 2.

### 3.3. Calibration of SRM−Tissue Partition by Dissipation Modelling (PRC)

**Figure 3:**
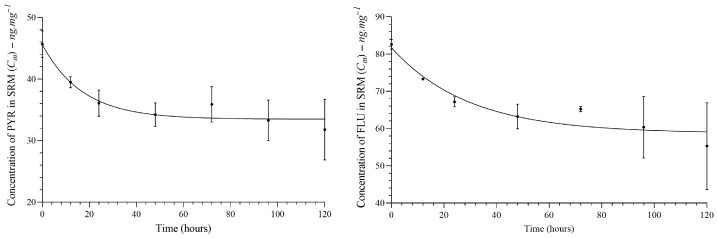
Analysis of PAH congeners diffusion between silicon membranes and fish tissue. The plateau was reached at time > 30 h for PYR and time > 40 h for FLU.

All PAH congeners diffused from SRM to fish tissue, which showed a lipid content of 4.5%. The diffusion of PAHs from SRM to fish tissue showed a model close to pseudo-first-order kinetics. See Figure 3. More details can be found in the Supplementary Material (See Figure S5).

**Figure 4:**
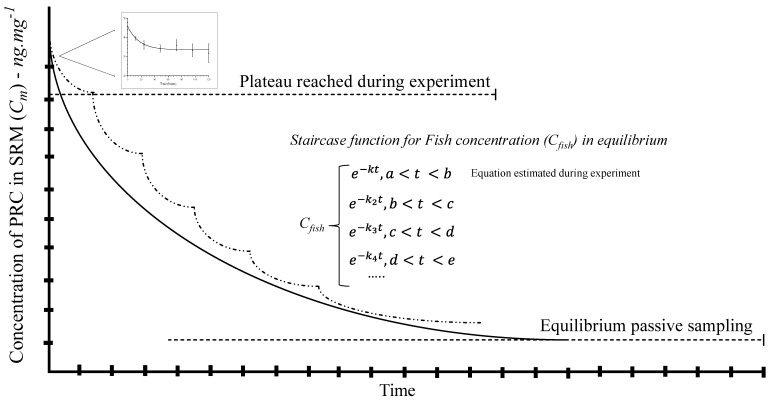
Complete equilibrium of passive sampling in fish tissue exposed to SRM. The figure shows a staircase function to integrate different plateaus in a single equation for the first kinetic order to reach the equilibrium. The small graph obtained was fitted into the first plateau. The dash line shows the segments or steps during the staircase function.

According to the mathematical model, delivery is constant, and the time to reach 50% of congeners partitioned in SRM–fish tissue (plateau) is presented in Table 2. However, the low lipid content (4.5%) in carp may decrease the efficiency of the diffusion process from SRM. Thereby, the equilibrium may not be ensured, but the plateau for a part of the staircase function was found. See Figure 4.

According to Rusina and colleagues, the relocation of SRM should be carried out to reach the pollutant diffusion at equilibrium. Thus, the PAHs bioconcentrated by lean fish tissue may diffuse with higher affinity to SRM in a single exposure. Usually, the SRM has shown strong affinity for nonpolar substances (*log K_ow_* > 4) in short times (days) [24]. However, more lipophilic substances (*log K_ow_* > 6) may take a long time to reach an equilibrium in silicone materials [23]. Therefore, the lipophilic range of PAHs may indicate a favorable diffusion between lean fish tissue and SRM. The favorable diffusion between *fl* and silicone materials has been previously applied for human body burden analysis [25].

Thereby, the calculus presented in Table 3 is based on the first plateau during the kinetic analysis as a small part of the staircase function. Relocation was not carried out because it is not an ethical protocol for in vivo fish, as presented in the next section. Both experiments (tissue partition and in vivo fish implantation) had to be carried out similarly for comparative kinetics. A higher amount of PAHs was found in SRM, and the percentages found in fish tissues reached values lower than 30%.

The *K_sf_* values were estimated by applying Equations (5) and (6). According to the *log K_sf_* presented in Table 3, the PAH congeners FLU and PYR showed stronger affinity to SRM than to fish tissue (values close to their *log K_ow_*). The *K_u_* for each one of the PAH congeners was estimated according to Equation (2). The values of Ku are presented in Table 3. The ratio between *K_e_* and *K_u_* shows faster SRM-PAH sorption than with PAH–fish tissue sorption (200 times higher). Therefore, the partition of those substances, such as FLU and PYR, in implanted fish may lead to faster silicon sorption or clearance from tissue, which indicates that this polymer takes an important role in long-term fish biomonitoring by passive sampling implantation.

### 3.4. Fish In Vivo Bio-Model

The carp was cultured under continuous monitoring parameters, such as temperature, dissolved oxygen, conductivity, total dissolved solids (TDS), and pH, to ensure optimal fish growth. See Supplementary Material for more details (See Figure S6c). The fluxes were higher than 2 L. min^−1^. The average values were plotted for the controlled parameters (Section S6). The water conditions showed a pH value of 8.2, conductivity of 36.5 mS cm^−1^, dissolved oxygen (DO) of 10.1 mg L^−1^, total TDS of 19.1 ppm, and temperature of 20 °C. Values showed to be appropriate for carp culture—more details can be found in the Supplementary Material (See Figure S6d).

The individual carp reached the stage of anesthesia 4, which is characterized by total loss of equilibrium (total loss of muscle tone), slow and regular opercular movements, and a loss of spinal reflexes [26]. More details are presented in the Supplementary Materials (See Figure S7b). The anesthesia process was continuously monitored to prevent overdosing or deeper stages of anesthesia. The induction of anesthesia and the time for recovery are presented in Table 4.

According to Table 4, lower deviation and lower %RSD were found in the induction and recovery time for individuals of carp. Values are related to low differences in weight in experimental fish, which were 1000.9 ± 90 g. The effects of anesthesia in fish depend on environmental conditions like the temperature of the water and its composition. On the other hand, the weight and physiological stage of fish individuals play an important role in the process of anesthesia [27]. For this study, environmental conditions were homogeneous since all fish came from the same pond, and water for anesthesia was taken from it.

Regarding individual conditions, all fish were adult common carp with low variable weights and sizes. Larger fish usually have longer induction times and shorter recovery times [27]. In this study, differences in weight and size of fish are not significantly different, but these parameters influence the times of induction of anesthesia and recovery [27]. However, we monitored the anesthesia stage to apply an appropriate anesthesia dose for induction (Table 4). The anesthesia in the batch process allowed for complete anesthesia to reduce the pain during surgery. According to Ferreira, the anesthesia is absorbed through the gills and skin of fish (e.g., scaleless fish with well-vascularized skin), which enables the high anesthesia stage [28].

### 3.5. Surgery for SRM Implantation in Dead Fish

**Figure 5:**
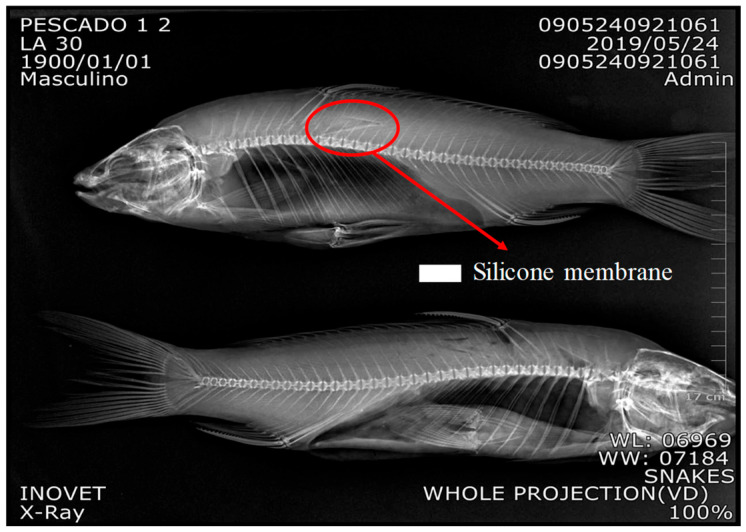
Shows that SRM was in a shallower place, but in contact with fish tissue. More details in Supplementary Materials (See Figure S8).

The incision for SRM implantation was initially tested in dead fish to gain more skills during surgery. More details are presented in Sections S7 and S8 (See Figure S7c Surgery procedures and Figure S8 Training for fish surgery using dead fish). The SRM was successfully implanted in tissue at a depth of 6 mm. See Figure 5 X-Ray in carp.

The *fl* in carp was 4.5%, and upon reaching equilibrium, less than 30% of PAHs had diffused into the tissue. The extent of equilibrium increased with increasing fish *fl*. For instance, previous research shows that the complete dissipation of PAHs in salmon (*Salmo salar*) (15% *fl*) is three times higher than in carp [14]. However, in this report by Rusina, the relocation of SRM was carried out to find the equilibrium at a low lipid amount in fish. In our work, the relocation was avoided due to ethical considerations during in vivo experiments. The low percentage delivered for PAHs partition may be associated with low lipid content in carp (partition processes between SRM and fish tissue). Additionally, the low temperature for experiments (10 °C) may decrease physical transferences based on the Arrhenius equation.

### 3.6. Surgery for Silicone Implantation In Vivo Fish

**Figure 6:**
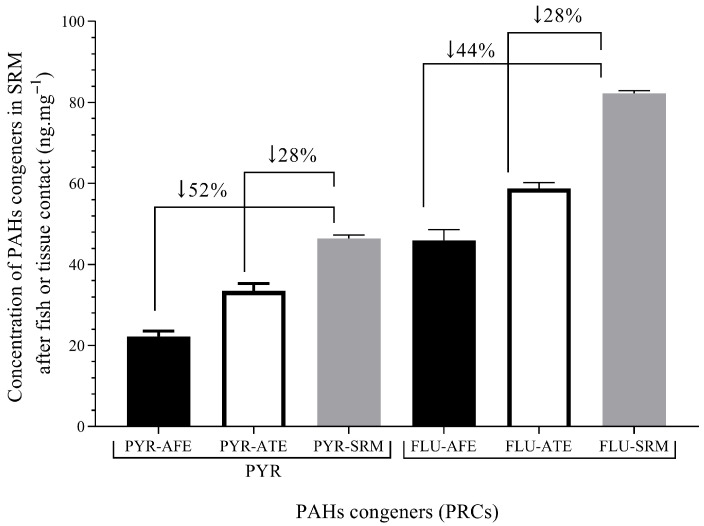
Levels of PAH congeners in SRM after fish or tissue exposure. PYR/FLU after fish exposure (PYR-AFE/FLU-AFE)—48 h exposure; PYR/FLU after tissue exposure (PYR-ATE/FLU-ATE) and PYR/FLU levels in SRM (PYR-SRM/FLU-SRM)—120 h exposure. The values show the percentage of decreasing levels in SRM after fish or tissue exposure. PYR-AFE and FLU_AFE (*n* = 4). The arrows indicate the groups analyzed by their percentages differences.

The SRM was implanted in vivo in carp after anesthesia for 48 h at a depth of 6 mm, close to the pectoral fin, following protocols presented before. The deuterated PAHs were not detected in silicon blank membranes implanted in control fish. This proves that the fish was not previously contaminated with waterborne PAHs, and previous bioconcentration had not occurred, at least at levels detectable with SRM. The carp were obtained from fish farms where they were cultured using high-quality water, reducing previous pollutant exposure. On the other hand, differences in the diffusion of deuterated PAHs between in vivo experiments and fish fillets were found. See Figure 6.

The changes in deuterated PAHs levels spiked in SRM (*N_o_*/*N_i_*) indicate that lipophilic substances, such as those used, like PRCs, may diffuse between SRM and fish tissue, which may be applied in partition processes. The percentage of PAHs in SRM showed higher values of diffusion in the in vivo fish experiment, which indicated partition and elimination processes due to metabolism.

The fish tissue exposure and in vivo fish exposure were tested against the control SRM, which is the spiked membrane without exposure. Thereby, differences between experiments in fish tissue (fillets) and in vivo fish were found. Thereby, the percentage of deuterated PAHs diffused from SRM to in vivo fish was 1.6 times higher than from SRM to fish fillet. The difference may also be related to fish biotransformation [29]. Although tissue decay may affect the diffusion, no changes in tissue were found because the low temperature was ensured during experiments. A similar temperature for fish culture was maintained (10–20 °C), and thus our experiments indicated partition and elimination processes due to metabolism. Therefore, both processes were integrated by applying equation 6 to estimate the levels of PYR and FLU in in vivo fish tissue. The parameters such as *K_e_* and *K_sf_* were taken from Table 3, while the *C_sm_* for PAH congeners were taken from Table 2. Results for in vivo fish tissue concentrations are presented in Table 5.

The PYR reached a 10% higher concentration than FLU in fish tissue during in vivo implantation. According to Figure 3, PYR reached a faster plateau than FLU in the SRM-fish tissue experiment. This substance shows a lower *log K_ow_* than FLU, which means a lower affinity for SRM. Additionally, the FLU shows a higher *log K_ow_*, and then this substance is more minimally eliminated than PYR by organisms, as shown in Table 3 (lower *K_e_* than PYR).

### 3.7. The Implication of the Implantation of SRM in Fish for the Uptake and Fugacity Study

The SRM has been preliminarily applied for fish tissue burden analysis of salmon, carp, and wild pikeperch (*Sander lucioperca*). This analysis has been carried out as a control for PCB levels allowed by EU regulation for freshwater fish fillets intended for consumption [14]. However, considering the ecotoxicology assessment of fish exposure is another important issue for understanding anthropogenic pollution effects. Although in vivo implantation takes an important role for uptake and fugacity of POPs, in vivo sampling may need to be combined with in vitro tissue-based measurements since not all organisms are amenable to implantation and because of calibration purposes [15]. Thus, the methodology in this study included fish tissue and in vivo implantation. The PRC procedure may be developed in the future to assess equilibrium or steady-state conditions between the organism and the polymeric phase.

Previously, Allan and colleagues estimated a possible relationship between *fl* and the potential fugacity [15]. In our study, we found that the low *fl* carp may represent a higher fugacity, which was evidenced by the inverse process under which a low amount diffused to the fish from SRMs. Similarly, fugacity has been assessed in humans. For instance, Allan and coworkers have focused on the use of silicone prostheses analysis for evaluating the human burden of POPs and fugacity, whose results have agreed with the bioaccumulation in other compartments such as serum and breast milk, which represent a promising matrix for the biomonitoring of nonpolar and non-ionic pollutants in humans [25]. Similarly, the in vivo fish implantation of SRM may represent future challenges for understanding pathways and real-level exposures of biota and fugacity. Although the equilibrium between fish tissue and SRM can take a long-time during implantation, the amount diffused to SRM during in vivo passive sampling can be used for fugacity analysis.

For that, implanted fish should be set free into water bodies under controlled conditions for analyzing bioaccumulation processes. In this paper, we presented some procedures for SRM–fish tissue studies to be applied to in vivo monitoring and a glimpse of kinetic parameters for interchange and elimination processes by using the dissipation modelling in SRM as the calibration method for passive sampling. As previously presented, the low *fl* reduces the dissipation of PRC from SRM to fish tissue, which does not allow it to reach the equilibrium. In this aspect, higher values of *K_sf_* mean a more favorable POPs fraction in SRM.

Although this study was limited to PAHs analysis, results may be extended to some HOCs and POPs with similar lipophilic properties (Similar *log k_ow_*). In this sense, the PAHs are relevant pollutants due to their sources and atmospheric deposition, which triggers a huge distribution in ecosystems [5,30].

## 4. Conclusions

The SRM implantation may be applied for uptake of POPs and fugacity studies of PAHs in fish with low *fl*, and relocation may be a method to address the higher lipid content in tissue analysis. However, an in vivo test required an unethical process with relocation (more surgeries). Our experiment showed a single compartmental model, and elimination was included, as known fish metabolized PAHs.

The *fl* should be considered for the equilibrium time because during monitoring using implanted fish with low values of *fl*, the pollutants prevail in the SRM phase. In contrast, in fish with higher *fl*, the pollutants will remain in fish tissue. Finally, the fish clearance should be included in the equation because this affects the equilibrium during passive sampling. Maybe the implantation in fish with higher *K_e_* will require lower time exposure because metabolism affects the pollutant distribution.

## Figures and Tables

**Table 1:** Validation parameters for the quantification of PAHs in SRMs. Retention time (*tR*), limit of detection (LOD), limit of quantification (LOQ), and the r-squared (*r*^2^).

PAHs	tR	LOD (μg·L^−1^)	LOQ (μg·L^−1^)	Linearity Range (μg·L^−1^)	*r* ^2^
FLU	20.7	8.8	25.1	25–500	0.998
PYR	21.1	9.3	25.7	25–500	0.999

**Table 2:** Concentration of PAHs in SRM (*n* = 3).

PAH Congener	Concentration in SRM (ng·mg^−1^)
FLU	81.74 ± 2.48
PYR	45.62 ± 1.38

**Table 3:** Parameters for fish tissue burden analysis.

PAHs	t_50_ (hours)	k_e_	K_u_	$C_{sm}^{\infty }$ (Plateau)	C_sm_ (ng.mg^−1^)	C_fit_ (ng.mg^−1^)	*log* K_sf_
FLU	21.6	0.03418	0.00017	58.72	81.74	0.00042	5.28
PYR	11.6	0.05868	0.00024	33.48	45.62	0.00022	5.31

t_50_: Half-time, *k_e_*: Elimination constant, *K_u_*: Uptake constant, *C_sm_*: Concentration in silicon membrane, *C_fit_*: Concentration in fish tissue, and *K_sf_*: Partition between silicon-fish.

**Table 4:** Monitoring of anesthesia stage in carp (CF). *n* = 5.

Experimental Carp	Time for Induction of Anesthesia (min)	Time for Recovery (min)
CF_1	4.1	8.2
CF_2	3.1	12.35
CF_3	2.5	10.15
CF_4	2.46	10.15
CF-5	3.26	10.35
Deviation	0.7	1.5
Average	3.1	10.2
% RSD	21.7	14.4

**Table 5:** Fish tissue burden.

PAHs Congeners	Concentration in Fish Tissue ng.mg^−1^
FLU	0.00034 (RSD 3.23)
PYR	0.00038 (RSD 4.44)

## Data Availability

The original contributions presented in this study are included in the article/Supplementary Material. Further inquiries can be directed to the corresponding author.

## Supplementary Materials

The following supporting information can be downloaded at: https://zenodo.org/uploads/18247497. Figure S1: Spiking using a Bach system (a). Extraction of SRM using a dialysis protocol. Table S1: Calculus for the expected concentration in SRM. Figure S2: Chromatogram for PAH congeners separation by GC/MS. Figure S3: Analysis by GC/MS for identification of PAHs in SRM samples. Figure S4: Curve calibration for Fluoranthene (FLU) and Pyrene (PYR). Figure S5: Fish fillet for partition coefficients estimation. Figure S6a: Tank and water fluxes for carp culture. Figure S6b: Tank for carp culture. Figure S6c: Analysis of physicochemical parameters. Figure S6d: Average and variation of physicochemical parameters for carp culture. Figure S7a: Carp under anesthesia protocol. Figure S7b: Carp under anesthesia protocol. Figure S7c: Surgery procedures. Figure S8: Training for fish surgery using dead fish.

## Abbreviations

The following abbreviations are used in this manuscript:

AF	Accumulation Factor
BAF	Bioaccumulation Factor
CF	Carp (*Cyprinus carpio*)
Cfit	Concentration in Fish Tissue
Csm	Concentration in Silicone Rubber Membrane
DO	Dissolved Oxygen
FLU	Fluoranthene
GC/MS	Gas Chromatography–Mass Spectrometry
HOCs	Hydrophobic Organic Compounds
Ke	Elimination Rate Constant
Ku	Uptake Rate Constant
Ksf	Silicone Rubber Membrane–Fish Tissue Partition Coefficient
*log K_ow_*	Logarithm of the Octanol–Water Partition Coefficient
LOD	Limit of Detection
LOQ	Limit of Quantification
PAHs	Polycyclic Aromatic Hydrocarbons
POPs	Persistent Organic Pollutants
PRCs	Performance Reference Compounds
PYR	Pyrene
RSD	Relative Standard Deviation
SIM	Selected Ion Monitoring
SRM	Silicone Rubber Membrane
t_50_	Time to Reach 50% of Equilibrium
TDS	Total Dissolved Solids
